# Mining geographic variations of Plasmodium vivax for active surveillance: a case study in China

**DOI:** 10.1186/s12936-015-0719-y

**Published:** 2015-05-27

**Authors:** Benyun Shi, Qi Tan, Xiao-Nong Zhou, Jiming Liu

**Affiliations:** School of Information Engineering, Nanjing University of Finance & Economics, Wenyuan Road, Nanjing, 210003 China; Key Laboratory of Symbolic Computation and Knowledge Engineering of Ministry of Education, Jilin University, Changchun, 130012 China; Department of Computer Science, Hong Kong Baptist University, Waterloo Road, Kowloon Tong, Hong Kong; National Institute of Parasitic Diseases, Chinese Center for Disease Control and Prevention; Key Laboratory of Parasite and Vector Biology, MOH; WHO Collaborating Center for Malaria, Schistosomiasis and Filariasis, Shanghai, 200025 China

**Keywords:** Geographic variation, Space-time model, Markov chain Monte Carlo, Active surveillance

## Abstract

**Background:**

Geographic variations of an infectious disease characterize the spatial differentiation of disease incidences caused by various impact factors, such as environmental, demographic, and socioeconomic factors. Some factors may directly determine the force of infection of the disease (namely, explicit factors), while many other factors may indirectly affect the number of disease incidences via certain unmeasurable processes (namely, implicit factors). In this study, the impact of heterogeneous factors on geographic variations of *Plasmodium vivax* incidences is systematically investigate in Tengchong, Yunnan province, China.

**Methods:**

A space-time model that resembles a *P. vivax* transmission model and a hidden time-dependent process, is presented by taking into consideration both explicit and implicit factors. Specifically, the transmission model is built upon relevant demographic, environmental, and biophysical factors to describe the local infections of *P. vivax*. While the hidden time-dependent process is assessed by several socioeconomic factors to account for the imported cases of *P. vivax*. To quantitatively assess the impact of heterogeneous factors on geographic variations of *P. vivax* infections, a Markov chain Monte Carlo (MCMC) simulation method is developed to estimate the model parameters by fitting the space-time model to the reported spatial-temporal disease incidences.

**Results:**

Since there is no ground-truth information available, the performance of the MCMC method is first evaluated against a synthetic dataset. The results show that the model parameters can be well estimated using the proposed MCMC method. Then, the proposed model is applied to investigate the geographic variations of *P. vivax* incidences among all 18 towns in Tengchong, Yunnan province, China. Based on the geographic variations, the 18 towns can be further classify into five groups with similar socioeconomic causality for *P. vivax* incidences.

**Conclusions:**

Although this study focuses mainly on the transmission of *P. vivax*, the proposed space-time model is general and can readily be extended to investigate geographic variations of other diseases. Practically, such a computational model will offer new insights into active surveillance and strategic planning for disease surveillance and control.

## Background

Disease surveillance systems play important roles in continuously monitoring the occurrence of an infectious disease at different geographic locations [1,2]. From the perspective of spatial epidemiology, the dependence or autocorrelations of disease incidences among nearby locations can be analysed from historical spatial-temporal disease incidences [3]. Accordingly, risk maps of the disease can be generated using appropriate spatial interpolation methods [4]. However, in reality, the natural transmission of an infectious disease can be potentially caused and affected by many impact factors, including but not limited to environmental, demographic, socioeconomic, behavioural, genetic, biophysical, and other risk factors [5-8]. Specifically, some factors may directly determine the risk of infection of the disease, namely, *explicit* factors, while many other factors may indirectly affect the disease incidences via certain unobservable processes, namely, *implicit* factors. In view of this, it would be desirable and essential to systematically assess the integrated impact of heterogeneous factors on the geographic variations of disease incidences [9,10]. By doing so, public health authorities can efficiently and effectively perform active surveillance and control by means of strategically planning and utilizing their limited resources.

Technically speaking, many methods have been proposed to analyse complex spatial-temporal distributions of disease incidences, and determine multiple impact factors underlying disease transmission. On the one hand, statistical analysis on different types of impact factors can produce risk maps of an infectious disease with respect to vectors [11], reservoirs [12], and human cases [13]. However, pure statistical analysis methods (e.g., spatial regression methods) are limited in exploring the real dynamics of disease transmission underlying the observed disease incidences. On the other hand, by systematically integrating various impact factors, various disease transmission models have been incorporated into the spatial statistics of infectious disease. Different from statistical analysis, disease transmission models can explicitly describe the underlying epidemiological process from the perspective of transmission mechanism. Taking the vector-borne diseases as an example, starting from the Ross model [14], a variety of differential equation models with different levels of complexity have been proposed to investigate the roles of different factors [15]. For example, Shi *et al.* have adopted a spatial transmission model to investigate the underlying disease transmission networks among different locations [16]. Unfortunately, due to the intrinsic complexity of disease transmission dynamics, there are still some other factors, the effects of which still cannot be explicitly interpreted.

**Fig. 1:**
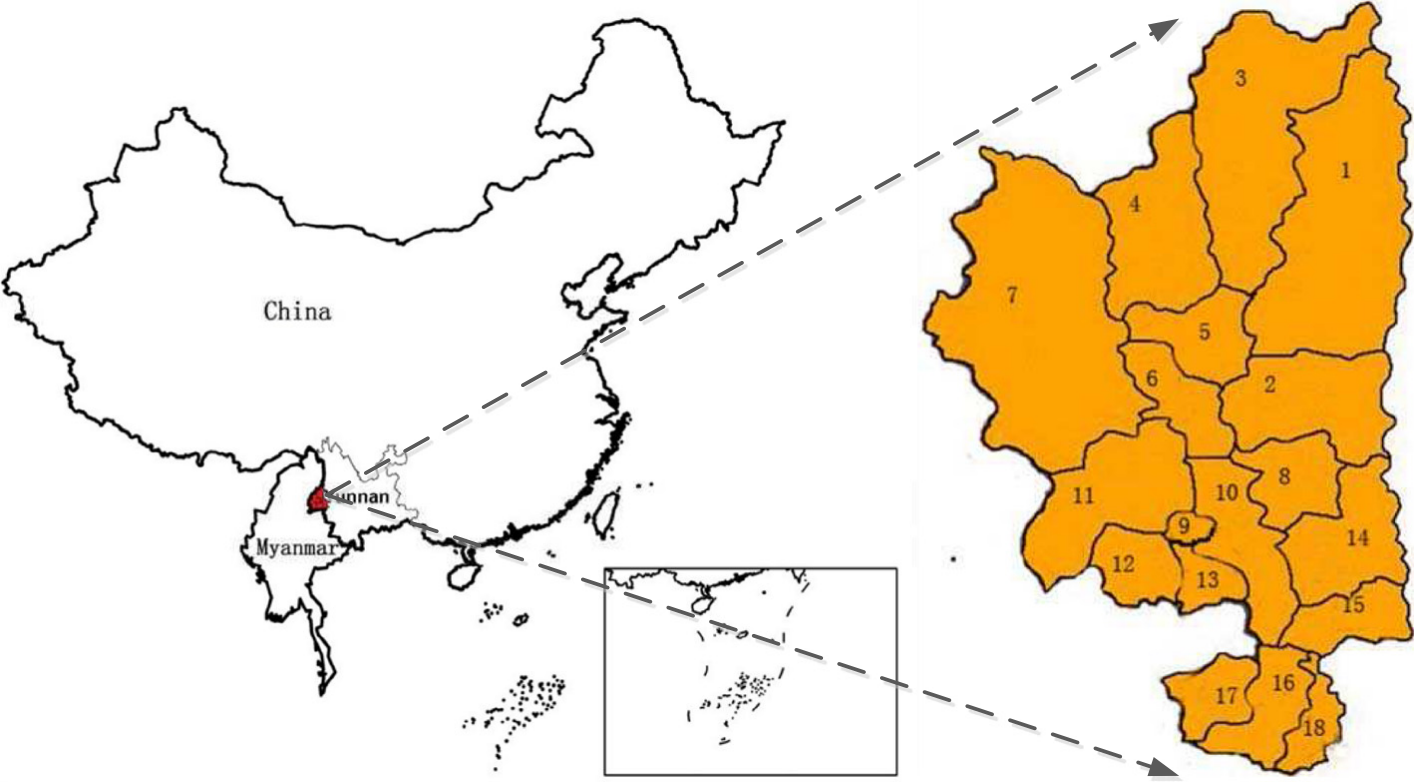
An illustration of the geographic locations of the 18 towns in Tengchong, Yunan province, China. The towns are marked in red, which are located near the national border between China and Myanmar.

This paper focuses on geographic variations of malaria incidences among 18 towns in Tengchong county, Yunnan province, China (see Fig. 1). The IDs and names of these towns are listed in Table 1. One reason that malaria is chosen as a case study lies in that it is one of the most serious and deadly infectious diseases all over the world, especially in developing countries [17,18]. In China, Yunnan province was ranked the first for the number of reported malaria cases, and the second for the incident rate of the disease from 1999 to 2004 [19]. While for Tengchong county in Yunnan province, all 18 towns have been experiencing high *Plasmodium vivax* transmission in the past years, with annual incidence rate higher than 1 per 10,000 [20,21]. With respect to the malaria elimination in Tengchong, it has been suggested by public health policy makers and practitioners that active surveillance would be an efficient strategy. Compared with passive surveillance (i.e., patients come to public health agencies for diagnosis and treatment), active surveillance aims to timely discover malaria infections through actively conducting on-the-spot investigation. However, in practice, active surveillance are extremely cost-expensive and time-consuming, which require massive experienced public health workers. So far, very few experienced workers are available, particularly in remote and underdeveloped regions in China. For instance, in Tengchong’s Centers for Disease Control (CDC), no more than five full-time workers are available to perform or coordinate the active surveillance for about 167 thousands households that are distributed in a wide area of more than five thousands square kilometres [22].

**Table 1:** **The IDs and names of the studied 18 towns in Tengchong, Yunnan province, China**

ID	Name	ID	Name	ID	Name
1	Jietou	2	Qushi	3	Mingguang
4	Ruidian	5	Gudong	6	Mazhan
7	Houqiao	8	Beihai	9	Heshun
10	Tengyue	11	Zhonghe	12	Hehua
13	Qingshui	14	Mangbang	15	Wuhe
16	Puchuan	17	Xinhua	18	Tuantian

Another important reason is that the situations of *P. vivax* transmission in Tengchong is complicated: first, researchers have shown that environmental factors (e.g., temperature and rainfall) have a significant impact on the population growth of mosquitoes, as well as their biological cycles [23,24]. Accordingly, due to the suitable climate in Tengchong, the force of infection of *P. vivax* to human being in individual towns varies depending on the dynamically changing environmental factors and its demographic profiles (e.g., human population size). Second, it was reported that the proportion of imported cases of *P. vivax* in China in 2011 is about 62.9 % [21], where the imported cases are defined as malaria infections whose origin can be traced to an area outside the country. While in Yunnan province, a large number of malaria incidences are imported from Myanmar due to cross-border economic activities [19,25]. Moreover, evidences have shown that the frequency of the cross-border activities is highly related to socioeconomic profile of each individual town, such as average income per capita [8,26,27].

To investigate the underlying causes of geographic variations of *P. vivax* incidences in Tengchong, this paper focuses not only on the direct impact of environmental and demographic factors on *P. vivax* transmission in individual towns, but also the indirect impact of socioeconomic factors on the number of imported cases. To achieve this, the following three critical challenges are addressed:

- How can a computational model be built to systematically characterize the impact of both explicit and implicit factors on geographic variations of disease incidences?
- How can the impact of imported cases on geographic variations be assessed using various socioeconomic factors by taking into consideration human cross-border activities?
- What kinds of computational methods can be developed to quantify geographic variations by fitting model parameters to observed *P. vivax* incidences?

To tackle these challenges, a space-time model is presented by extending the idea of factor analysis, which has been extensively adopted to investigate spatial-temporal patterns of infectious diseases [28,29].

Specifically, the space-time model consists of a linear combination of a *P. vivax* transmission model and a hidden time-dependent process of a set of non-observed common factors. First, a malaria transmission model is built based on the notion of vectorial capacity (VCAP), which characterizes the *P. vivax* transmission potential based on dynamically changing temperature, rainfall, as well as population size in each individual town [30,31]. Then, socioeconomic factors are integrated into a hidden time-dependent process of a set of common factors, which help quantify the variations of different towns in terms of the number of imported cases. To quantitatively assess geographic variations of *P. vivax* incidences, a Morkov chain Monte Carlo (MCMC) simulation method is used to fit the proposed space-time model to the spatial-temporal *P. vivax* incidences [32,33].

To evaluate the performance of the proposed space-time model, experiments are first conducted on a set of synthetic data generated using predefined model parameters. The results show that the MCMC method can well estimate all model parameters. Then, a real-world study is carried out to investigate the geographic variations of *P. vivax* incidences among all 18 towns in Tengchong, Yunnan province, China. Model parameters are estimated by fitting the proposed model to monthly-reported *P. vivax* incidences from 2005 to 2010. Based on the estimated model parameters, the 18 towns are classified into several groups in terms of the impact of their socioeconomic factors on the number of imported cases. By doing so, public health authorities can strategically allocate their limited resources to specific groups of towns so as to improve the efficiency of active surveillance.

In summary, even through this study introduce the space-time model by taking *P. vivax* transmission in Tengchong as an example, the proposed model is not limited to analysing geographic variations of *P. vivax* incidences. Without loss of generality, it can also be extended to analyse spatial-temporal data series of other diseases.

## Methods

### A space-time model

Disease surveillance systems usually monitor disease incidences of different locations as a set of time series. Given the observed disease incidences of *N* locations during time period *t*=1,⋯,*T*, the spatial-temporal surveillance data at time *t* can be represented by a vector *y*_*t*_=(*y*_1*t*_,⋯,*y*_*Nt*_)^′^. With respect to malaria transmission in Tengchong, China, the number of *P. vivax* incidences of each individual town consists of two parts: one is local infections caused by the *P. vivax* transmission within the town, which can be explicitly modelled based on environmental and demographic factors; the other is imported cases caused by a hidden time-dependent dynamics (e.g., human cross-border activities), which can be implicitly affected by a set of socioeconomic factors. According to the study in [34], the space-time model can be defined as follows:

(1) $$\begin{array}{*{20}l} y_{t} &= u_{t} + \beta \cdot f_{t} +\epsilon_{t}, \hspace*{60pt} \epsilon_{t} \sim N(0,\Sigma) \end{array} $$

(2) $$\begin{array}{*{20}l} f_{t} &= \Gamma \cdot f_{t-1}+w_{t}, \hspace*{68pt} w_{t} \sim N(0,\Lambda) \end{array} $$

where *u*_*t*_ describes the epidemiological dynamics of local *P. vivax* transmission at time *t*, and *β*·*f*_*t*_ describes a hidden time-dependent dynamics of imported cases. Specifically, *u*_*t*_=(*u*_1*t*_,⋯,*u*_*Nt*_)^′^ represents the number of local infections at time *t*, *f*_*t*_ is an *m*-dimensional vector of common factors (i.e., the order of the factor model), and *β*=(*β*_(1)_,⋯,*β*_(*m*)_) is the *N*×*m* factor loading matrix. Each row of *β* describes the importance of common factors for a given town, while each column of *β* (i.e., *β*_(*i*)_) shows spatial dependence of different towns with respect to a specific common factor. In this paper, it is assumed that the values of common factors at time *t* depend only on those at time *t*−1, where the matrix *Γ* characterizes the time-dependent dynamics of the common factors. Finally, *Σ* and *Λ* are observational and time-dependent variations. For simplicity, it is also assumed that $\Sigma = diag({\sigma _{1}^{2}}, \cdots, {\sigma _{N}^{2}})$ and $\Lambda = diag\left ({\lambda _{1}^{2}}, \cdots, {\lambda _{N}^{2}}\right)$.

By fitting model parameters to spatial-temporal surveillance data, the main objective is to evaluate the impact of heterogeneous factors on geographic variations of *P. vivax* incidences.

### Epidemiological dynamics of malaria transmission

The notion of vectorial capacity (VCAP) is used to assess *P. vivax* transmission potential using environmental and demographic data, which is defined as “the number of potentially infective contacts an individual person makes, through vector population, per unit time [15].” The VCAP was adapted from the basic reproductive number calculated based on the Macdonald model [35]. In each town *i*, the value of VCAP is given by:

(3) $$ V_{i} = \frac{-(m_{i} {a_{i}^{2}})p_{i}^{n_{i}}}{\ln(p_{i})},  $$

where *m*_*i*_ represents the equilibrium mosquito density per person, *a*_*i*_ is the expected number of bites on human beings per mosquito per day, *p*_*i*_ is the probability of a mosquito surviving through one whole day, and *n*_*i*_ is the entomological incubation period of malaria parasites. Based on the study of Ceccato *et al.* [30], all these parameters are dependent on human population *P*_*i*_, as well as dynamically-changing temperature (*T*) and rainfall (*R*) in each individual town. Here, the detailed parameter descriptions and settings for calculating the VCAP of each individual town are shown in Table 2, which is adopted from the existing work [16]. As mentioned in [16], the values of relevant parameters are based on a certain degree of assumptions and estimates, and they could be adjusted when more accurate values are available.

**Table 2:** **The parameter descriptions and settings for calculating vectorial capacity**

Parameters	Descriptions	Values
Gonotrophic cycle length: *U*=0.5+*f*_*u*_/(*T*−*g*_*u*_)		
*f*_*u*_	The number of degree days needed for maturation	36.5 ([30])
*g*_*u*_	The threshold below which gonotrophic development ceases	9.9 ([30])
*T*	The average temperature of an individual town	MODIS ([40])
The probability of daily survival: *p*=*α* ^1/*U*^		
*α*	The proportion of vectors surviving each gonotrophic cycle	0.5 ([30])
Sporogonic cycle length: *n*=*f*_*n*_/(*T*−*g*_*n*_)		
*f*_*n*_	The number of degree days required for parasite development	105 ([24,48])
*g*_*n*_	The threshold below which parasite development ceases	18 °C ([30])
Human biting habit: *a*=*h*/*U*		
*h*	The human blood index	0.7 ([30])
The ratio of mosquitoes to human: *m*=10*R*/*P*		
*R*	The average rainfall of an individual town	TRMM ([41])
*P*	The human population in an individual town	Census ([22])

The table is adopted from the existing work [16]

Based on the relationship of VCAP and entomological inoculation rate (EIR), the number of infectious bites received per day by a human being can be estimated [31]. Accordingly, the number of local infections at time *t* can be calculated based on the number of infections at previous time *t*−1. The formulation is as follows:

(4) $$ u_{t} = \frac{-bcV_{t}y_{t-1}'y_{t-1}}{P_{i}}+ y_{t-1}I(1-r+bcV_{t}),  $$

where *b* represents the probability that a susceptible person becomes infected after being bitten by an infectious mosquito, *c* denotes the probability that an uninfected mosquito becomes infected after biting an infectious person, *r* is the human recovery rate, *I* is *N*×*N* identity matrix, and *V*_*t*_=(*V*_1*t*_,⋯,*V*_*Nt*_)^′^ is a vector of VCAP for different towns at time *t*. It should be noted that the model parameters *bc* and *r* will be estimated by fitting the proposed model to the spatial-temporal malaria incidences.

### Time-dependent dynamics of common factors

As in standard dynamic factor model [36], in this paper, Equation 2 describes the dynamics of *mindependent* common factors, where *Γ* is set to be *diag*(*γ*_1_,⋯,*γ*_*m*_). In doing so, the factor loading matrix *β* characterize geographic variations of disease incidences with respect to the set of common factors. In this paper, the *j*th column of *β* is modelled as a Gaussian random field (GRF), that is,

(5) $$ \beta_{(j)}\sim GRF\left(\mu_{j}^{\beta}, {\tau_{j}^{2}}R_{\phi_{j}}\right),  $$

where $\mu _{j}^{\beta }$ is *N*-dimentional mean vector, ${\tau _{j}^{2}}$ indicates the scale of spatial dependence, $R_{\phi _{j}}$ is a symmetric and positive definite covariance matrix. The element $R_{\phi _{j}}(l,k)$ can be used to reflect the range of spatial dependence in terms of geographic distances and socioeconomic factors. Specifically, (*l*,*k*)-element of the covariance matrix is given by $R_{\phi _{j}}(l,k) = \rho _{\phi _{j}}(s_{\textit {lk}})$, where $\rho _{\phi _{j}}(\cdot)$ is a correlation function and *s*_*lk*_ represents the spatial heterogeneity between towns *l* and *k* [34]. Here, the correlation function is assumed to be exponential, i.e.,

(6) $$ \rho_{\phi_{j}}(s_{lk}) = \exp\left(-{s_{lk}}/{\phi_{j}}\right),  $$

where *ϕ* can be generated from an inverse gamma distribution.

The spatial heterogeneity *S*={*s*_*lk*_}_*N*×*N*_ is defined as the Hadamard product of a geographic distance matrix *D* and a socioeconomic distance matrix *M*, i.e., *S*=*D*∘*M*, where *M* is given by the Cosine distances between different towns with respect to a list of *n* implicit impact factors *x*=(*x*_1_,⋯,*x*_*n*_). Therefore, each element in *M* can be calculated as follows:

(7) $$ M_{lk} = 1-\frac{x_{l} \cdot x_{k}}{\| x_{l}\|\cdot \| x_{k}\|} = 1- \frac{\sum_{i=1}^{n} x_{li}x_{ki}}{\sqrt{\sum_{i=1}^{n} x_{li}^{2}}\sqrt{\sum_{i=1}^{n} x_{ki}^{2}}},  $$

where *x*_*l*_ represents a vector of impact factors ofz location *l*. To generate *D*, geographic distances between the 18 towns in Tengchong are extracted using Google Maps API. Meanwhile, five socioeconomic factors are used to calculate the socioeconomic distance matrix *M*, they are: per capita arable land, per capita food production, per capita meat production, per capita government revenue, and personal income. Clearly, Equation 6 indicates that the pairwise covariance and hence dependence between any two towns decreases as the heterogeneity between them increases. It should be note that although only five socioeconomic factors are used in this paper, the calculation of spatial heterogeneity can be extended to involve more implicit factors.

### Inferring model parameters

In this section, an MCMC simulation method is presented to estimate model parameters by fitting the proposed space-time model to disease incidences data.

Mathematically, the space-time model can be reformulated in matrix notation as *y*=*u*+*Fβ*^′^+*ε*, where *y*=(*y*_1_,⋯,*y*_*T*_)^′^ is a *T*×*N* matrix, *u*=(*u*_1_,⋯,*u*_*T*_)^′^ is a *T*×*N* matrix, and *F*=(*f*_1_,⋯,*f*_*T*_)^′^ is a *T*×*m* matrix. The matrix *ε* is of dimension *T*×*N*, and follow a matrix-variate normal distribution, i.e., *ε*∼*N*(0,*I*_*T*_,*Σ*) [34]. Thus, given *m* number of common factors, the posterior probability of *y* can be calculated as follows:

(8) $$\begin{array}{@{}rcl@{}} p(y|F,\! \beta,\! \Theta) &=& \!(2\pi)^{-TN/2}|\Sigma|^{-T/2}\times \\  && \exp\!\left(\!tr\!\left(\!-\frac{\left(y\!-u\!-F\beta'\right)'\left(y-u-F\beta'\right)}{2\Sigma}\right)\right), \end{array} $$

where *Θ* consists of parameters in the time-dependent dynamics of common factors, i.e., $\sigma = ({\sigma _{1}^{2}}, \cdots,{\sigma _{N}^{2}})$, $\lambda = \left ({\lambda _{1}^{2}}, \cdots,{\lambda _{m}^{2}}\right), \gamma = \left (\gamma _{1}, \cdots,\gamma _{m}\right), \mu = \left (\mu _{1}^{\beta }, \cdots,\mu _{m}^{\beta }\right), \tau = \left ({\tau _{1}^{2}}, \cdots, {\tau _{m}^{2}}\right), \phi = \left (\phi _{1}, \cdots, \phi _{m}\right)$, as well as parameters in the epidemiological dynamics of *P. vivax* transmission, i.e., *bc* and *r*. Accordingly, the joint posterior distribution of (*F*,*β*,*Θ*) is given by:

(9) $$\begin{array}{@{}rcl@{}} p\left(F, \beta, \Theta|y\right) &\propto & \prod_{t=1}^{T} p\left(y_{t}|f_{t}, \beta, \sigma\right)p(bc)p(r)p(f_{0}) \\  &\times & \prod_{t=1}^{T} p\left(f_{t}|f_{t-1}, \lambda, \gamma\right) \\  &\times & \prod_{j=1}^{m} p\left(\beta_{(j)}| \mu_{j}^{\beta}, {\tau_{j}^{2}}, \phi_{j}\right)p\left(\gamma_{j}\right)p\left(\mu_{j}^{\beta}\right)p\left({\tau_{j}^{2}}\right)\\  &\times & p\left(\phi_{j}\right) \prod_{i=1}^{N} p\left({\sigma_{i}^{2}}\right) \prod_{i=1}^{N} p\left({\lambda_{i}^{2}}\right), \end{array} $$

where the prior information of the model parameters (*F*,*β*,*Θ*) will be discussed in detail in the Results section.

To simultaneously estimate the model parameters, an MCMC simulation method is developed. The procedure of the method is as follows: First, all independent model parameters *Θ*(0)=(*σ*,*λ*,*γ*,*μ*,*τ*,*ϕ*,*bc*,*r*,*f*_0_) are initialised based on their prior distributions. Then, the values of factor loading matrix *β*(0) and the values of common factors *f*_1_ are generated based on Equation 6 and Equation 2, respectively. By doing so, the posterior distribution *p*(*F*(0),*β*(0),*Θ*(0)|*y*) can be estimated based on Equation 9. For each iteration, new values of parameters *Θ*^∗^ will be generated from an adaptive proposal distributions *q*(*Θ*^∗^|*Θ*) [32,33]. Accordingly, new values of *F*^∗^ and *β*^∗^ will be calculated. All the new values *F*^∗^, *β*^∗^ and *Θ*^∗^ will be accepted with probability:

(10) $$ \min \left(1, \frac{p\left(F^{*},\beta^{*},\Theta^{*}|y\right)q\left(\Theta|\Theta^{*}\right)}{p\left(F,\beta,\Theta|y\right)q\left(\Theta^{*}|\Theta\right)} \right).  $$

After a total number of *M* iterations, the statistics of the factor loading matrix *β* and other model parameters can therefore be analysed. The detailed method is shown in Algorithm 1.



## Results

### Simulated study: the evaluation of the MCMC simulation method

To evaluate the performance of the MCMC method, a synthetic dataset is simulated based on the proposed space-time model with a set of predefined model parameters. Then, the ability of the method to estimate model parameters is assessed by treating the predefined model parameters as ground-truth values.

#### Data generation

To simulate the synthetic dataset, the geographic environment and the parameters of the proposed space-time model are set as follows:

- Similar to the study in [34], *N*=25 locations are uniformly allocated in a two-dimensional square [0,1]×[0,1], that is, the longitudes and latitudes of individual locations are (0.20,0.20), (0.20,0.40), ⋯, (1.00,0.80), (1.00,1.00), respectively.
- After surveying existing literatures about the dynamics of malaria transmission, epidemiological parameters are set to be *bc*=0.007 and *r*=0.05.
- The observational and the time-dependent variations are set to be *Σ*=*diag*(0.02,0.02,0.02) and *Λ*=*diag*(0.02,0.03,0.01), respectively. Moreover, the matrix *Γ* is set to be *Γ*=*diag*(0.60,0.40,0.30).
- Without loss of generality, it is assumed that there are three common factors (i.e., *m*=3). The factor loading matrix *β* is generated from a Gaussian process of exponential correlation function with *ϕ*=(0.15,0.40,0.25). In other words, $R_{\phi _{j}(l,k)} = \exp (-d_{\textit {lk}}/\phi _{j})$.
- The value of $\mu _{j}^{\beta }$ is only relevant to distance in the simulated experiments. Accordingly, it is reasonable to set $\mu _{j}^{\beta }= X\mu _{j}$, where *X*=(1_*N*_,*Longitude*_*N*_,*Latitude*_*N*_), and *μ*_1_=(5,5,4)^′^, *μ*_2_=(5,−6,−7)^′^, and *μ*_3_=(5,−8,6)^′^. The scalar *τ* is set to be *τ*=(1.00,0.75,0.56).

The objective is to evaluate whether the proposed MCMC simulation method can help estimate the time-dependent diagonal matrix *Γ*, the scalar *τ*, the epidemiological parameters *bc* and *r*, as well as the number of common factors *m*.

#### Parameter settings

The model parameters are estimated by fitting the space-time model to the generated data using the proposed MCMC algorithm. Specifically, the following prior distributions are adopted with respect to each parameter in the MCMC method:

- The observational and time-dependent variations follow inverse gamma distribution, i.e., *σ*^2^∼*IG*(0.01,0.01) and *λ*^2^∼*IG*(0.01,0.01).
- The parameters in *Γ* are assumed to follow a normal distribution, i.e., *γ*_*i*_∼*N*(0.5,1).
- The initial values of common factor *f*_0_ is set to be *f*_0_=(0.6,0.4,0.3).
- According to literature review, the epidemiological parameters *bc* and *r* are assumed to follow uniform distributions, where *bc*∼*U*(0.0036,0.01248) and *r*∼*U*(0.02222,0.11110).
- The factor loading matrix is modelled as a Gaussian random field, i.e., $\beta _{j} \sim N(\mu _{j}^{\beta }, {\tau _{j}^{2}} R_{\phi _{j}})$, where $\mu _{j}^{\beta }$ is a known hyperparameter and follows a normal distribution with mean value equal to true value and variance equal to 25, the scale of spatial dependence ${\tau _{j}^{2}}$ follows an inverse Gamma distribution, i.e., ${\tau _{j}^{2}} \sim IG(1, 0.75)$, and the prior distribution of *ϕ*∼*IG*(2,*b*) for *b*= max(*S*)/(−2 ln(0.05)) and max(*S*) is the largest element for all *s*_*lk*_ (see [37,38], for more detail).

#### Simulation results

**Fig. 2:**
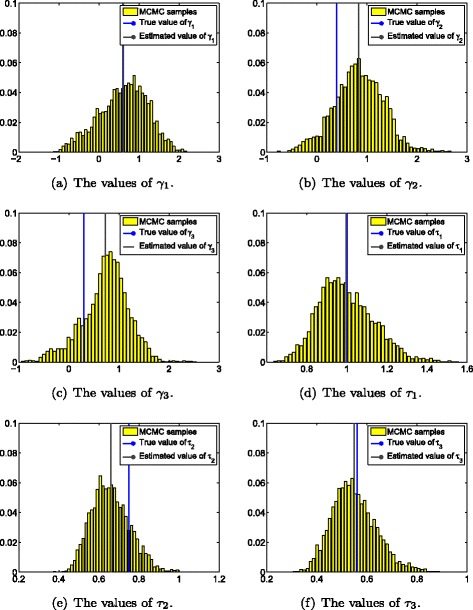
The estimates of model parameters *Γ* and *τ* using the proposed MCMC simulation method.**a**, **b**, **c** The estimated mean values of *γ*_1_, *γ*_2_, and *γ*_3_ (black lines) and their corresponding true values (blue lines); **d**, **e**, **f** The estimated mean values of *τ*_1_, *τ*_2_, and *τ*_3_ (black lines), and their corresponding true values (blue lines). **a** The values of *γ*_1_. **b** The values of *γ*_2_. **c** The values of *γ*_3_. **d** The values of *τ*_1_. **e** The values of *τ*_2_. **f** The values of *τ*_3_.

**Fig. 3:**
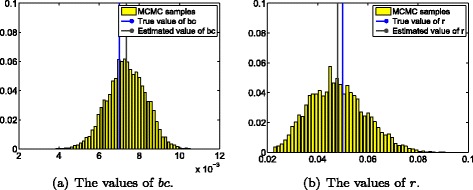
The estimates of epidemiological parameters *bc* and *r* using the proposed MCMC simulation method.**a** The estimated mean values of *bc* (the dark lines) and its true value (the blue line); (**b**) The estimated mean values of *r* (the dark lines) and its true value (the blue line). **a** The values of *bc*. **b** The values of *r*.

The MCMC algorithm is run for 100,000 iterations, and the posterior inference is built upon the last 80,000 draws. Figure 2 shows the estimated parameters of *γ* and *τ* using the proposed MCMC simulation method, while Fig. 3 demonstrates the estimated values of epidemiological parameters *bc* and *r*. In all these figures, the true value of each parameter is illustrated using a blue line, while the estimated mean value is shown using a dark line. The detailed values and their corresponding 95 % credible intervals are shown in Table 3. It can be observed that all estimated mean values are very close to their true values (Figs. 2 and 3), and the estimated mean values of all model parameters are within their corresponding 95 % credible intervals (Table 3).

**Table 3:** **The estimates of model parameters and their 95 % credible intervals**

	*γ*_1_	*γ*_2_	*γ*_3_	*τ*_1_	*τ*_2_	*τ*_3_	*bc*	*r*
True values	0.60	0.40	0.30	1.00	0.75	0.56	0.0070	0.0500
Mean	0.61	0.80	0.73	0.997	0.66	0.55	0.0074	0.0479
Variance	0.60	0.49	–0.47	0.14	0.096	0.09	0.0009	0.0113
5 %	–0.48	–0.02	–0.13	0.79	0.51	0.41	0.0058	0.0304
50 %	0.66	0.84	0.78	0.98	0.65	0.54	0.0074	0.0473
95 %	1.54	1.61	1.44	1.24	0.83	0.71	0.0089	0.0679

Besides the model parameters, another important factor needs to be determined is the value of *m* in the time-dependent dynamics of common factors (i.e., the order of the factor model). In this simulation study, several models with up to five common factors (i.e., *m*=2, 3, 4, and 5) are tested with respect to four measurements. They are two measurements about fitting errors (i.e., the mean absolute error (*MAE*) and the mean square error (*MSE*)) and two criteria about model selection (i.e., the Akaike information criterion (*AIC*) and the Bayesian information criterion (*BIC*)), where $MAE = \frac {1}{NT}\sum _{i=1}^{N}\sum _{t=1}^{T} |y_{\textit {it}}-\hat {y}_{\textit {it}}|$, $MSE = \frac {1}{NT}\sum _{i=1}^{N}\sum _{t=1}^{T} (y_{\textit {it}}-\hat {y}_{\textit {it}})^{2}$, *AIC*=2*m*−2 ln(*L*), and *BIC*=*m* ln(*n*)−2 ln(*L*). Here, *L* is the value calculated by Equation 8, and *n* is the number of observed data.

Table 4 shows the performance of the simulated studies with respect to models with different number of common factors. It can be found that *m*=3 reaches the best performance in terms of above-mentioned four measurements, which is exactly the number of common factors used for generating the synthetic dataset.

**Table 4:** **The effects of the number of common factors**

*m*	*MAE*	*MSE*	*AIC*	*BIC*
2	0.42	0.36	3072.33	3265.35
3	0.19	0.07	718.04	1004.56
4	0.23	0.09	1114.45	1494.46
5	0.25	0.11	1456.99	1930.50

In summary, the above results suggest that the MCMC simulation method can well estimate the values of the model parameters and the order of the factor model.

### Real-world study: the *P. vivax* transmission in Tengchong, Yunnan, China

This section focuses on the investigation of the effects of various impact factors on the geographic variations of *P. vivax* incidences among 18 towns in Tengchong, Yunnan province, China.

#### Data collection

With respect to monthly malaria incidences from 2005 to 2010, different towns show different temporal patterns. There are two major reasons: first, due to the environmental and demographic heterogeneity of these towns, malaria transmission potential in each individual town is different. Second, due to the socioeconomic heterogeneity, human cross-border activities in individual towns are different, which may affect the number of imported malaria incidences. The following data are involved in constructing the space-time model.

- *Malaria incidences*. The reported cases of *P. vivax* infection are collected from the China Information System for Disease Control and Prevention, which cover all the 18 towns in Tengchong from 2005 to 2010 [39].
- *Temperature and rainfall*. The temperature and rainfall data of Tengchong from 2005 to 2010 are collected to estimate the *P. vivax* transmission potential for individual towns. For the temperature, the Moderate Resolution Imaging Spectroradiometer (MODIS) is used to estimate near-surface air temperature [40]. For the rainfall, the Tropical Rainfall Measuring Mission (TRMM) product is used to estimate daily precipitation [41].
- *Population size*. The population size of each town is based on the sixth national census of China in 2010 [22].
- *Geographic distances*. The geographic distances between individual towns are identified as the shortest road distances using Google Maps API.
- *Socioeconomic factors*. Suggested by public policy makers and practitioners, five typical socioeconomic factors are adopted to characterize socioeconomic heterogeneity of the studied towns from 2005 to 2010, they are, per capita arable land, per capita food production, per capita meat production, per capita government revenue, and personal income. All these data are collected from Tengchong Statistics Bureau. It should be noted that many other factors from heterogeneous data sources can also be involved into the calculation of matrix *M* in the proposed space-time model.

#### Parameter settings

To estimate model parameters, the same prior distributions as that in simulated study are used for parameters *σ*^2^,*λ*^2^,*γ*,*bc*,*r*,*τ*^2^ and *ϕ*. The other two parameters *f*_0_ and $\mu _{j}^{\beta }$ are set as follows:

- The initial values of *f*_0_ are drawn from a normal distribution, i.e., *f*_0_∼*N*(1,1).
- The factor loading matrix is modelled as a Gaussian random field, i.e., $\beta _{j} \sim N(\mu _{j}^{\beta }, {\tau _{j}^{2}} R_{\phi _{j}})$. Here, $\mu _{j}^{\beta }$ follows a normal distribution with the same mean and variance as that of *y*_*t*_−*u*_*t*_ for all *t*, where the values of *u*_*t*_ is calculated using randomly generated *bc* and *r* from their prior distributions.

#### Simulation results

**Fig. 4:**
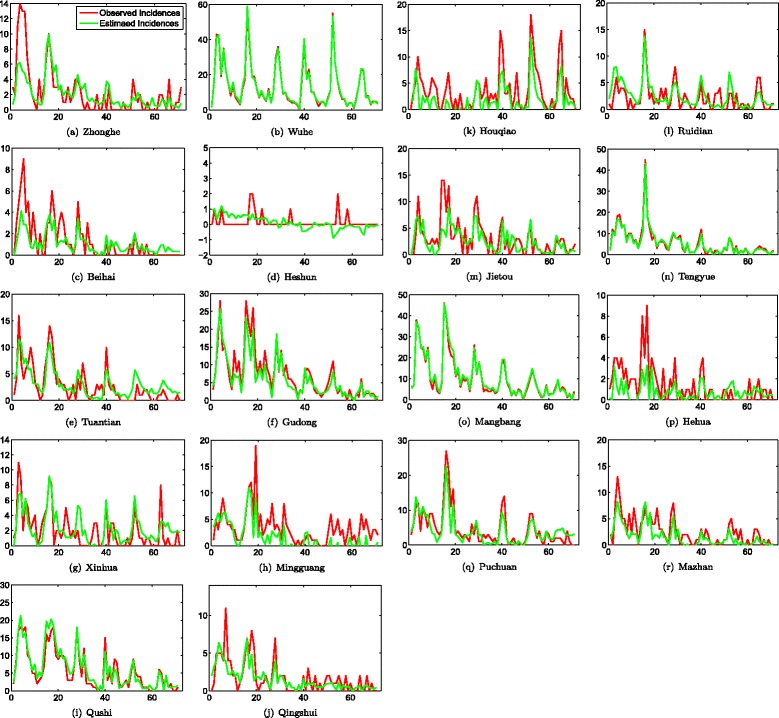
The observed and estimated numbers of *Plasmodium vivax* incidences of the 18 towns in Tengchong, Yunnan province, China, by month from 2005 to 2010. The red lines correspond to the observed numbers of *Plasmodium vivax* incidences, while the green lines show the estimated numbers of *Plasmodium vivax* incidences based on the proposed space-time model. (**a**) Zhonghe, (**b**) Wuhe, (**c**) Beihai, (**d**) Heshun, (**e**) Tuantian, (**f**) Gudong, (**g**) Xinhua, (**h**) Mingguang, (**i**) Qushi, (**j**) Qingshui, (**k**) Houqiao, (**l**) Ruidian, (**m**) Jietou, (**n**) Tengyue (**o**) Mangbang, (**p**) Hehua, (**q**) Puchuan and (**r**) Mazhan.

The MCMC algorithm is run for 100,000 iterations with a burn-in of the first 20,000 runs. First, the appropriate number of common factors *m* is incrementally evaluated in terms of the four measurements, i.e., *MAE*, *MSE*, *AIC*, and *BIC*. It can be found that better performances can be achieved when *m*=5. Figure 4 shows the fitting results of monthly *P. vivax* incidences of the 18 towns in Tengchong, from 2005 to 2010. The red lines correspond to the observed numbers of incidences, while the green lines show the estimated numbers of incidences based on the proposed space-time model. It can be observed that for most towns, the proposed model preforms very well in terms of fitting the real-world observations, except for certain special towns, such as the town Heshun in Fig. 4d. The possible reason is that *P. vivax* incidences in Heshun are temporally sparse. Therefore, historical malaria incidences play limited roles in estimating future incidences, in other words, the time-dependent process will dominate the final estimation. However, such misestimate is tolerable in real world because the number of *P. vivax* incidences in these towns is relative small.

**Fig. 5:**
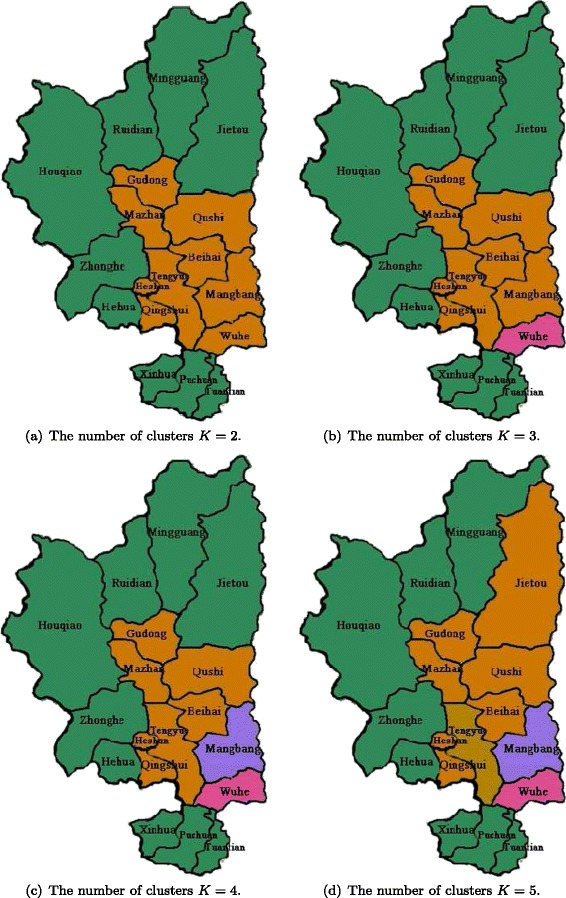
The classification results of the 18 towns in Tengchong, Yunnan province, China. Different colors to represent different clusters. (**a**) The number of clusters *K*=2; (**b**) The number of clusters *K*=3; (**c**) The number of clusters *K*=4; and (**d**) The number of clusters *K*=5.

According to the definition of factor loading matrix *β*, each row of *β* represents the importance of common factors for a given town, and each column of *β* shows spatial dependence among different towns. In this case, each column of *β* can be treated as an “attribute” of individual towns so as to classify the 18 towns based on the impact of their “attributes” on geographic variations of *P. vivax* incidences. Table 5 shows the estimate of the factor loading matrix *β* with the number of common factors *m*=5. Along this line, the well-known *K*-means algorithm is adopted to do classification based on the estimated factor loading matrix *β*. Figure 5 demonstrates the classification results of the 18 towns by setting *K*=2, 3, 4, and 5, where different colors represent different clusters. It can be found that when *K*=2, some adjacent towns are grouped into one cluster (e.g., the brown cluster and the green cluster in Fig. 5a), which means that geographic distances may dominate variations of malaria incidences. This is inline with the analysis of certain spatial statistics methods, such as the a smoothed surface map in [16]. Specifically, several towns adjacent to Tengyue is classified into the same cluster (i.e., the brown cluster in Fig. 5). The reason may be that Tengyue is the center of Tengchong county, and have relatively better economic status. Peoples in these towns may seldom travel to high risk region in Myanmar. As the value of *K* increases, some special towns (i.e., Wuhe and Mangbang) will gradually separate from brown cluster, possibly due to the integrated impact of socioeconomic factors. By doing so, active surveillance and targeted intervention strategies can be implemented for groups of towns based on the amount of available resources, which may significantly improve the effectiveness and efficiency of malaria control and elimination.

**Table 5:** **The estimate of the factor loading matrix** ***β*** **with the number of common factors** ***m*** **= 5**

Town ID	Factor 1	Factor 2	Factor 3	Factor 4	Factor 5
1	–0.03	1.52	–1.22	–0.03	–0.78
2	–0.78	3.02	–0.06	–0.46	–1.09
3	2.15	1.16	–0.36	–0.52	0.19
4	0.55	–0.17	0.50	0.75	–0.17
5	–3.16	1.73	2.28	0.13	–0.33
6	–0.87	0.27	1.07	0.15	–0.19
7	–0.52	–1.21	–0.91	2.08	0.18
8	–0.44	0.41	0.35	–0.09	–0.37
9	–0.03	0.10	0.17	–0.19	–0.09
10	1.35	–0.31	4.11	–0.05	–1.77
11	0.47	0.60	0.43	0.06	0.24
12	0.61	0.43	–0.72	0.20	0.35
13	–0.50	0.32	0.95	–0.19	–0.32
14	–0.37	1.92	1.91	2.01	2.88
15	1.05	0.59	–0.78	4.81	–2.39
16	2.48	0.36	–0.11	0.96	0.55
17	0.54	0.85	–0.48	0.31	–0.45
18	0.22	0.40	0.06	0.67	0.31

## Discussion

Data mining and spatial statistics methods play essential roles in understanding spatial-temporal patterns of disease incidences, which can provide valuable information for disease surveillance and control. First, local clusters or hot spots of disease transmission can be identified through geostatistical analysis on the time series of disease incidences, where targeted intervention strategies can be applied to improve the efficiency of disease control. For example, researchers have adopted the SaTScan software to detect local malaria clusters based either on confirmed malaria cases [42], or other related impact factors [43]. Second, spatial dependence between different locations can be quantified to reveal the relationships between the severity of an infectious disease and its relevant impact factors. For example, Osei and Duker have studied the spatial dependence of *Vibrio cholera* prevalence on open space refuse dumps [44]; Gemperli *et al.* have investigated environmental and age dependence of malaria transmission in West and Central Africa [45]. Third, incidences at unobserved locations can be estimated using appropriate spatial interpolation methods based on confirmed incidences at observed locations. For example, Kriging linear spatial interpolation method has been adopted to visualize geographic and temporal trends in rotavirus activity in the United States [46]. Regarding the above-mentioned problems, most existing methods have focused solely on the impact of several typical factors. While the aim of this paper is to *systematically* modelling geographic variations of disease incidences by taking into consideration various impact factors from heterogeneous data sources.

Factor analysis is one kind of statistical methods to systematically describe a large number of correlated variables using a potentially small number of unobserved variables (i.e., factors). Generally speaking, the main purpose of factor analysis on spatial epidemiology is to either reduce the overall dimension of observations at each geographic location, or describe temporal dynamics of all locations using a small set of common factors [34,36]. Different from existing studies, the observations of disease incidences is univariate (i.e., the spatial-temporal distribution of disease incidences) and the main focus is to investigate the impact of heterogeneous impact factors on geographic variations of disease incidences. In this paper, the space-time model is one of the first attempts to study both explicit and implicit factors by integrating the epidemiological dynamics of disease transmission and the time-dependent dynamics of unobserved common factors.

Although the experimental results have shown that the proposed space-time model can perform well in fitting to the reported spatial-temporal *P. vivax* incidences in Tengchong, it should be noted that the model can still be able to be generalized in the following ways: first, in this paper, it is assumed that the values of common factors *f*_*t*_ at time *t* depend on those at previous time *f*_*t*−1_. In reality, the duration of time window should be justified based on the real-world situations, such as the incubation period of the infectious diseases. Second, the entries in matrix *Γ* is constant throughout the paper. Theoretically, it can be generalized to involve time-dependent entries of *Γ* such that dynamic patterns of common factors (e.g., seasonal patterns) can be investigated. Third, in the MCMC method, the number of common factors is incrementally evaluated. While in the future, a customized reversible jump MCMC method [47] can be utilized to learn the appropriate value of *m*. Lately, it can be observed from the experimental results (e.g., Fig. 4d) that when the *P. vivax* incidences is temporally spare, the proposed model cannot well fit the observed numbers of incidences. Therefore, some specialized methods should be developed when the observed disease incidences in most geographic locations are temporally sparse.

Last but not the least, the proposed space-time model is a linear combination of a disease transmission model and a hidden time-dependent process. In the future, various data mining methods can be involved to design more complicated space-time model by explicitly revealing the impact of other heterogeneous factors. Moreover, in addition to mining geographic variations of disease incidences, the proposed model can also be extended to conduct the following problems:

- *Incidence forecasting*. Based on the estimated model parameters, the proposed model can also be used to forecast disease incidences in the near future. Mathematically, the *h*-steps ahead predictive density *p*(*f*_*T*+*h*_|*f*_*T*_,*β*,*Θ*) can first be learned. Then, *p*(*y*_*T*+*h*_|*f*_*T*+*h*_,*β*,*Θ*) can be estimated.
- *Spatial interpolation*. Based on spatial interdependence, disease incidences in unobserved locations may be estimated by analysing locations with similar values of impact factors. To achieve this, new inference methods need to be proposed to estimate unobserved rows in factor loading matrix *β*.

All these issues are worth further pursuing so as to achieve effective and efficient disease surveillance and control.

## Conclusions

In this paper, a space-time model is presented to investigate geographic variations of disease incidences by taking into consideration two types of impact factors: one is the explicit factors that can directly affect the dynamics of malaria transmission; the other is the implicit factors that may indirectly affect the number of imported cases. Without loss of generality, the model is implemented to investigate geographic variations of *P. vivax* incidences among 18 towns in Tengchong, Yunnan province, China. Specifically, the notion of vectorial capacity is adopted to model the *P. vivax* transmission potential with respect to environmental and demographic factors. Meanwhile, the spatial heterogeneity of different towns is characterized in terms of their geographic distances and five types of socioeconomic factors. Based on the space-time model, these factors may result in geographic variations of *P. vivax* incidence through the time-dependent dynamics of a set of common factors. To estimate the model parameters, an MCMC simulation method is used by fitting the model to the spatial-temporal disease incidences. A synthetic study is carried out to assess the ability of the MCMC method in estimating model parameters. Then, the proposed model is applied to conduct a real-world study on investigating geographic variations of *P. vivax* incidences among the 18 towns in Tengchong. It is expected that the computationally obtained methods and results may offer public health authorities with further insight into, as well as new tools for, active surveillance and control of infectious diseases.

## Abbreviations

MCMC: Markov chain Monte Carlo
CDC: Centers for disease control
VCAP: Vectorial capacity
GRF: Gaussian random field
MAE: Mean absolute error
MSE: Mean square error
AIC: Akaike information criterion
BIC: Bayesian information criterion
